# Describing objectively measured physical activity levels, patterns, and correlates in a cross sectional sample of infants and toddlers from South Africa

**DOI:** 10.1186/s12966-017-0633-5

**Published:** 2017-12-22

**Authors:** Alessandra Prioreschi, Soren Brage, Kylie D. Hesketh, Jill Hnatiuk, Kate Westgate, Lisa K. Micklesfield

**Affiliations:** 1 MRC/WITS Developmental Pathways for Health Research Unit, Department of Paediatrics, School of Clinical Medicine, Faculty of Health Sciences, University of Witwatersrand, Johannesburg, South Africa; 2 MRC Epidemiology Unit, University of Cambridge, Cambridge, UK; 3 Institute for Physical Activity and Nutrition, Deakin University, Geelong, Australia

**Keywords:** Infant, Physical activity, Television viewing, Accelerometery, South Africa

## Abstract

**Background:**

Physical activity is considered to have health benefits across the lifespan but levels, patterns, and correlates have not been well described in infants and toddlers under the age of two years.

**Methods:**

This study aimed to describe objectively and subjectively measured physical activity in a group of South African infants aged 3- to 24-months (*n* = 140), and to investigate individual and maternal correlates of physical activity in this sample. Infants’ physical activity was measured using an Axivity AX3 wrist-worn accelerometer for one week and the mean vector magnitude was calculated. In addition, mothers reported the average amount of time their infant spent in various types of activities (including in front of the TV), their beliefs about infants’ physical activity, access to equipment in the home environment, and ages of motor development milestone attainment. Analysis of variance (ANOVA) and pair-wise correlations were used to test age and sex differences and associations with potential correlates.

**Results:**

There were significant age and sex effects on the distribution of time spent at different physical activity intensities (Wilks’ lambda = 0.06, *p* < 0.01). In all cases, the trend was for boys to spend more time in higher intensity physical activity and less time in lower intensity activity than girls; and for time spent in higher intensity activities to be higher in older children. Time spent outside was higher in boys, and this reached significance at 18-months (F = 3.84, *p* = 0.02). Less concern around floor play was associated with higher physical activity at 12-months in females only (*p* = 0.03, *r* = 0.54), and no other maternal beliefs were correlated with physical activity. The majority (94%) of children were exceeding TV time recommendations. When controlling for age and sex, overall TV time was positively associated with BMI z-score (β=0.01, *p* = 0.05).

**Conclusion:**

This study is the first to show sex and age differences in the patterns of physical activity, and to report on objectively measured and maternal reported physical activity and sedentary behaviour in the first two years of life in South Africa infants. Infants and toddlers should be provided with as many opportunities to be active through play as possible, and TV time should be limited.

## Background

Physical activity has beneficial effects on health at all stages of the lifecourse. Data from higher income countries has shown that physical activity in early childhood (0–4 years) has beneficial effects on current and future health, and that sedentary behaviour may have a detrimental influence on body composition, which subsequently influences health outcomes [1–3]. Physical activity in the early years has also been shown to be beneficial for cognitive and motor development, and emotional and physical well-being [2]. Further, in a systematic review increased screen time has been shown to be associated with weight gain in children under age two [4]; and a meta-analysis of current evidence has shown that rapid weight gain in the first two years of life is associated with increased adiposity in later childhood [5]. However, there is a paucity of data describing objectively measured physical activity and sedentary behaviour at a very young age i.e. in the first two years of life [6].

The first two years of life have been shown to be critical determinants of adult behaviours and health risks [7], yet in South Africa no studies have objectively measured physical activity and sedentary behaviours in this period [7]. According to survey data, South Africans present with high sedentary time and low physical activity levels from a young age [8]. A high prevalence of overweight is already apparent in young children (ranging from 11%–14% in children aged 6 years [9], with 20% of toddlers at age two presenting with overweight [8]). Similar to international findings, approximately half of South African children are meeting age specific physical activity recommendations [9]. Internationally, studies have shown that participation in sedentary behaviors (mainly television (TV) viewing) is already higher than recommended in the first two years of life [4, 10, 11]; and similarly excessive sedentary time (73% of the day) is apparent in South African children as young as 4 years old [12].

Correlates of activity levels in the first two years of life have not been extensively described [6]. A systematic review of the cross sectional correlates of physical activity in preschool children (aged 2–5 years) conducted in 2008 showed that being male, spending more time outdoors, and having an active parent, were correlated with being more active [13]. A more recent systematic review of longitudinal observational studies and controlled trials conducted in 2016 showed that predictors of increased physical activity in 0–6 year olds included parental monitoring of their children’s activity, maternal role modeling; and there was some inconsistent evidence that boys had higher physical activity than girls [14]. This systematic review reported that very few studies had been conducted in healthy children under age two, or in developing countries (with none conducted in Africa), however recently studies have been conducted measuring physical activity response to dietary treatment or supplementation in undernourished toddlers in Africa [15, 16]). Studies on infants and toddlers have consistently shown age to be associated with increased sedentary behaviour and with more time spent in higher intensity physical activity [17–20], yet it is likely that developmental stage (rather than chronological age) will impact on ability to participate in certain types and intensities of activity, thus presenting a bidirectional relationship between developmental stage and physical activity [21]. Furthermore, maternal factors such as mother’s TV time and physical activity were associated with toddler sedentary time and physical activity, respectively [17, 18, 20]. Mother’s education level (higher), race (minority), and the home environment; and time toddlers spent being active with their mother and with other toddlers, have also been correlated with toddler sedentary time and physical activity respectively [17–19]. In toddlers who were walking (aged 12- to 36-months), boys were more active than girls [20].

The need to measure and examine levels of physical activity and sedentary time in the first two years of life is clear. Furthermore, correlates of these behaviours should be examined in order to intervene effectively. Preliminary data and data from older children suggest that the home environment and mother’s behaviours, and child sex and age (and/or developmental stage) are important correlates of physical activity and sedentary behaviour. Therefore this study aims to 1) describe levels and patterns of physical activity and sedentary behaviour in a group of South African infants/toddlers aged 3- to 24-months using objective and subjective data, and 2) investigate and describe individual and maternal correlates of objectively measured physical activity.

## Methods

### Participants and procedures

Participants (convenience sample of 152 mother and child pairs) were recruited from the Chris Hani Baragwanath Academic Hospital, MRC/Wits Developmental Pathways for Health Research Unit in Soweto, South Africa. Soweto is over 200km^2^ in size with over 1.2 million inhabitants. Fathers in Soweto often have little to no involvement in childcare [22], and therefore only mothers were considered in this study. In most cases, mothers had been recruited from the Chris Hani Baragwanath Academic Hospital (which is the largest public hospital in South Africa and is thus likely to provide a sample of mothers representative of the Soweto community) for participation in a pregnancy and infancy study (M130905) at the research unit, and were invited telephonically to participate in the current study if their child was within 2 weeks of 3-, 6-, 12-, 18-, or 24-months of age (information obtained via pregnancy study birth records). For the purposes of this paper, children of all ages from this sample will be referred to as “infants”. Participants were excluded if infants had any diagnosed developmental abnormalities that may affect normal movement or development. Mothers were required to read and sign informed consent and assent documents for themselves and on behalf of their infant, and were free to withdraw from the study at any time. Ethical approval for this study was provided by the University of the Witwatersrand Human Research Ethics Committee (M150632).

Participants were required to visit the research unit twice. At the first visit all demographic information was collected, questionnaires completed, measurements taken, and infants were fitted with an accelerometer and mothers were provided with a sleep diary to complete for their infant. At the second visit, one week later, participants were required to return the accelerometers and sleep diaries. If participants were unable to return the devices, researchers collected the devices from participants’ homes.

### Outcome measures

#### Physical activity

Infants’ physical activity was objectively measured using an Axivity AX3 wrist worn accelerometer (Axivity Ltd., Newcastle-upon-Tyne, UK). These monitors allow for transparent data processing analysis due to the open-source software platform and unforced sampling of raw measurement data [23, 24]. Furthermore, they are small enough to be worn on the wrist of infants from 3 months. Monitors were initialised (using the open source OmGui software, Open Movement, UK) to capture triaxial acceleration data at 100 Hz with a dynamic range of +/−8 g. The monitors were worn in a specially designed fabric band (Open Lab, Newcastle, UK), which is waterproof, breathable, flexible and safe. This band was designed and piloted by the authors and Open Lab, and has been successfully used and tested for feasibility in a South African sample of infants (manuscript under review [25]). Mothers were instructed not to remove the band from their infant’s wrist for a seven-day period. Following download (OmGui, Open Movement, UK), the raw acceleration data were autocalibrated to gravity, using methods described elsewhere [26]; and vector magnitude (expressed in mg) was calculated representing the magnitude of acceleration over the summarised time period [23]. A high-pass (0.2 Hz) and low-pass (20 Hz) frequency filter was then applied to the data in order to remove gravity, as well as high frequency noise [27]. Thus, the remaining data provided an approximation of acceleration due to human movement alone. Thereafter, non-wear was identified based on the deviation of each axis below 13 mg for >1 h, and removed [23]. Remaining data was summarised to generate average acceleration (mg), as well as time (hourly and weekly) spent in intensity thresholds (10 mg increments from 0 to 400 mg). During summation, non-wear periods were taken into account in order to minimize potential diurnal bias [28]. At least 45 h of wear time (equating to three days of at least 15 h data per day [29]) spread equally across the 24-h day (at least 4 h each day during morning, noon, afternoon, and night) were required for inclusion in the analysis.

### Correlates and covariates

#### Anthropometry and demographics

Mothers were asked to report their date of birth and their infants’ date of birth and sex. Infant age was calculated and categorised to the nearest month. Mothers’ height was taken to the nearest 1 mm using a wall-mounted stadiometer (Holtain, UK). Infant length was measured to the nearest 1 mm using an infantometer (Chasmors Ltd., UK). Weight of mothers and infants was measured to the nearest 0.1 kg using a digital scale (Dismed, USA). All anthropometry measurements were taken twice, by trained research staff according to standardised procedures, and the average of the two values was reported. Mother and infant body mass index (BMI) was calculated as (weight(kg)/height(m)^2^). Infant weights, lengths and BMIs were converted to age-specific z scores according to the 2006/2007 World Health Organisation (WHO) growth standards [30] using the WHO Anthro software [31]. Mothers were asked to report how many hours per day they looked after their infant, and were considered primary caregivers if they reported looking after their infant for 8 or more hours per day.

#### Questionnaire data

All questionnaires used in this study were based on previously developed and reliability tested items [19], which were then adapted for language and setting based on pilot work conducted on mothers from Soweto. These adaptations mainly consisted of rewording sections that were not well understood in a South African context (i.e.: “…a baby that is very active” was changed to “…a baby that moves a lot”).

#### Infant activities

Infant activities were assessed at 3-, 6-, 12-, and 18-months. Mothers reported the amount of time (minutes) their infant spent in various activities, on an average day. Activities were age-specific, but included tummy time (only 3- and 6-months), time spent playing games with an adult, time spent being physically active with the mother, time spent on the floor, time spent with other babies of a similar age, time spent with older toddlers or children, time spent outside, time spent in front of a television (TV), time spent strapped to the mother’s back (only 3-, 6- and 12-months), and time spent restrained (in a highchair, car chair, pushchair etc). Activities were pooled to determine total time restrained (i.e. sum of time spent strapped to mother’s back, time spent in a highchair, car chair, pushchair, or cot), and total time free to play (i.e. sum of tummy time, time spent playing games with an adult, time spent being physically active with mother, time spent on the floor, time spent with other babies of a similar age, time spent with older toddlers or children, time spent outside). All activities are presented as minutes per day. Maternal reported time that infants spent in front of a TV was extracted from this questionnaire and used as proxy for TV time in order to examine potential correlates thereof. During the measurement period, mothers were asked to complete sleep diaries recording the time at which their infant was put to bed at night, and the time at which they were picked up from bed in the morning in order to provide an estimate of time in bed. If sleep diary data were missing, values were imputed based on trends for other infants’ days and times.

#### Maternal belief questionnaire

Maternal beliefs about their infant’s physical activity, and attitudes and intentions around their infant’s physical activity and TV viewing, were assessed at 6- and 12-months using a 24-item questionnaire with a 4-point likert-type scale and response options: 1 = strongly agree to 4 = strongly disagree. Based on previous studies [19], 7 factor variables were created, including physical activity knowledge (7 questions examining the importance of physical activity for infant health and development), positive views about physically active children (3 questions examining maternal perceptions of active children), negative views about physically active children (4 questions examining maternal fears and concerns about physically active children), physical activity optimism (3 questions examining the anticipated ease of engaging children in physical activity), self-efficacy for promoting physical activity (3 questions examining mothers’ confidence for promoting physical activity), future expectations around infant’s physical activity and TV viewing (2 questions examining maternal expectations of infant’s future physical activity and TV viewing behaviours), and floor play concerns (2 questions examining perceptions of safety of floor play). Scores for each factor variable were then generated by averaging the item scores within each factor, where a higher score indicates lower agreement with the factor variable. The internal consistency in the present sample (mean Cronbach alpha = 0.60) for these factor variables was acceptable and comparable to previous studies [19], with only one factor variable (positive views about physical activity) scoring below 0.60.

#### Home environment questionnaire

Mothers of infants aged 3-, 6-, 12-, and 18-months were asked to report, using a 4 point likert-type scale, whether their child had access to, or was likely to have access to age-specific toys and equipment (such as balls, push toys, bicycles, etc) in the home or at nearby facilities within the next year. Each response was coded as “3” if mothers responded that they had the equipment already, “2” if they were very likely to get it within the next year, “1” if mothers responded they would possibly get the equipment in the next year, and “0” if mothers responded that they were unlikely to get the equipment within the next year. Thereafter a sum of the responses for each item was created, with a higher score indicating better access to equipment or intention to provide access, and a lower score indicating no access and/or less intention to provide access.

#### Developmental milestones

Mothers of infants aged 3-, 6-, 12-, and 18-months were asked to report whether their infant had attained specific motor development milestones according to the age of the child (based on the CDC recommendations: www.cdc.gov/ActEarly). Milestones included: holding up head at 3-months; sitting, crawling, and rolling to both sides at 6-months; crawling, pulling up to stand, and starting to walk at 12-months; and walking and running at 18-months. Milestones were recorded as attained/not attained. Thereafter, infants were categorised as either not yet mobile (this assumed all 3-month infants, as well as those aged 12-months who were not yet crawling); crawling (if 18-month infants were not yet walking/running they were presumed to be crawling); or walking/running (all infants aged 24-months were presumed to be walking/running). Assumptions were based on WHO reported ages for milestone attainment [32].

### Statistical analysis

All statistical analyses were conducted using Stata 13 for Mac. Participant characteristics and all questionnaire data were summarised by age and presented as mean (SD), n (%), or median (IQR). Since there is no consensus on intensity thresholds for sedentary, light, moderate, and vigorous activities in non-ambulatory infants (which made up the majority of the sample), objectively measured infant physical activity intensity distribution was presented as time spent in each systemic acceleration category from 0 to 400 mg by age and sex. Furthermore, hourly mean vector magnitude (diurnal patterns) of activity were compared between ages, sex, and developmental stages. These differences were tested using multivariate analysis of variance (ANOVA) through Wilks’ lambda test. Two-way ANOVAs, linear regressions, and students’ unpaired t-tests were used to test age and sex differences in the time spent in various activities as reported by mother, as well as for objectively measured physical activity. Thereafter, pairwise correlations were run by age for maternal variables (home environment, maternal BMI and age, maternal-reported infant activities, and maternal beliefs factor variables) on infant objectively measured physical activity. Regressions were also run for potential correlates of maternal reported infant TV time controlling for age and sex, and margins were calculated. Chi-squared tests were used to determine the proportion of infants meeting TV time guidelines according to developmental stage. A *p* < 0.05 was considered significant in all cases.

## Results

**Fig. 1:**
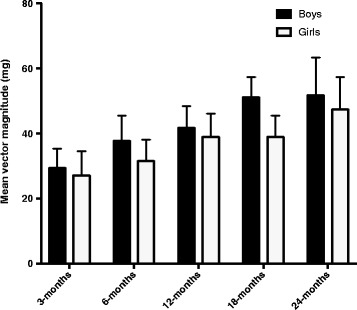
Mean volume of physical activity by age and gender

Of the 152 mother-infant pairs recruited for the study, six children did not have sufficient monitor wear time and were excluded from analyses. A further six infants lost their accelerometers or had missing data files. Participant characteristics for the remaining 140 participants with complete data are shown in Table 1. Although weight and length displayed were significantly higher between each consecutive age group except between 18- and 24-months (*p* < 0.01), there were no differences in BMI z-score by age (*p* = 0.61) or sex (*p* = 0.97), nor were there differences in weight (*p* = 0.14) or length (*p* = 0.47) between the boys and girls. Average reported time in bed was 10 h 18 min (range 7 h 29 min to 13 h 26 min), and did not differ significantly by age or sex (*p* = 0.09 and *p* = 0.91 respectively). Most mothers (96%) looked after their infants for ≥8 h per day. Boys were significantly more active than girls when comparing mean vector magnitude for the entire sample (*n* = 140) over the total wear period (41(11)mg vs 36(11)mg, *p* < 0.01), and this remained significant when categorising by age at 6-, and 18-months. Total average physical activity for boys ranged from 21 mg–83 mg; and for girls ranged from 17 mg–61 mg. Physical activity had a direct linear relationship with age (*p* < 0.01) as shown in Fig. 1. Neither weight (*p* = 0.86), length (*p* = 0.55), nor BMI z-score (*p* = 1.00) were associated with physical activity when controlling for age and sex.

**Table 1:** Characteristics of the study sample by age

	3mo (*n* = 32)	6mo (*n* = 31)	12mo (n = 31)	18mo (*n* = 18)	24mo (*n* = 28)
Infant sex (% boys)^a^	14 (44)	21 (68)	15 (48)	10 (56)	14 (50)
Infant weight (kg)^b^	5.8 (0.8)	7.7 (1.2)	9.0 (1.0)	9.9 (1.5)	10.8 (1.2)
Infant length (cm)^b^	58.7 (5.1)	65.0 (2.7)	72.9 (3.6)	79.3 (4.3)	82.3 (3.9)
Infant BMI z score^b^	0.2(2.5)	0.6 (1.6)	0.3 (1.3)	−0.3 (1.6)	0.2 (1.0)
Attainment of milestones at given age^a^					
*Holding head*	21 (66)	NA	NA	NA	NA
*Rolling*	NA	13 (42)	NA	NA	NA
*Sitting*	NA	16 (52)	NA	NA	NA
*Crawling*	NA	7 (23)	16 (52)	NA	NA
*Pulling up*	NA	NA	14 (45)	NA	NA
*Walking*	NA	NA	9 (29)	6 (33)	28 (100)
*Running*	NA	NA	NA	4 (22)	NA
Maternal BMI (kg/m^2^)^b^	28.5 (9.1)	27.8 (6.7)	29.5 (7.0)	27.5 (8.0)	27.3 (6.7)
Maternal age (years)^b^	27 (5)	31 (6)	29 (5)	32 (6)	29 (7)
Infant PA (mg)^c^	23 (17–28)	30 (24–34)	36 (30–39)	39 (35–45)	44 (37–51)
Home Environment (score/48)^b^	24 (8)	20 (4)	21 (5)	24 (5)	NA
Maternal Beliefs (score/4)^a^					
*PA Knowledge*	NA	1.4 (0.3)	1.4 (0.3)	NA	NA
*PA views*	NA	1.7 (0.5)	1.9 (0.6)	NA	NA
*Negative PA views*	NA	3.0 (0.5)	2.8 (0.6)	NA	NA
*PA optimism*	NA	1.4 (0.4)	1.5 (0.5)	NA	NA
*Future expectations*	NA	2.5 (0.8)	2.6 (0.7)	NA	NA
*Floor play concerns*	NA	3.0 (0.8)	3.0 (0.6)	NA	NA
*Self efficacy*	NA	1.4 (0.3)	1.5 (0.4)	NA	NA

*BMI* - body mass index, PA - physical activity. At 24-months, all toddlers were assumed to be walking although not specifically reported at this time-point

*NA* Indicates that data was not acquired at the specific age

^a^n(%)

^b^All data are presented as mean(SD)

^c^or median(IQR)

**Fig. 2:**
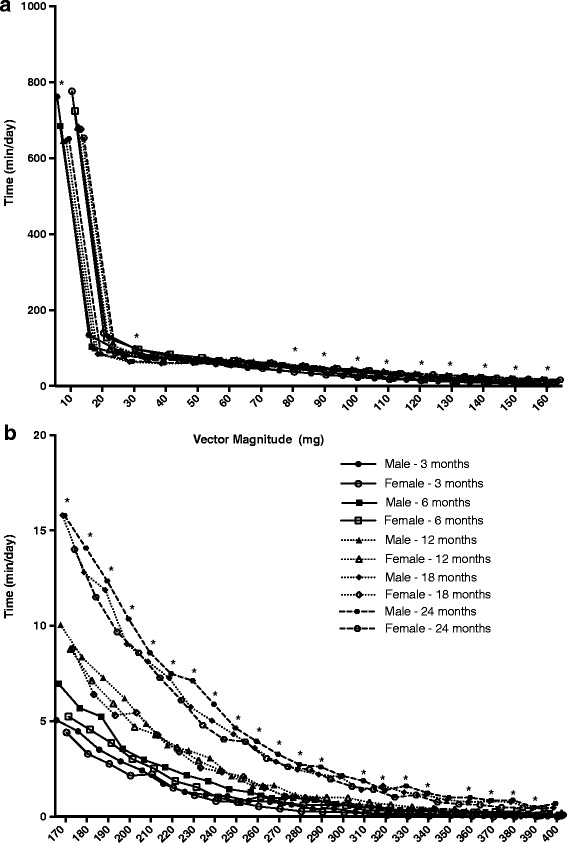
Distribution of time spent <160 mg (**a**) and >160 mg (**b**) between sex and ages. * Indicates significant difference between boys and girls, controlling for age (*p* < 0.05)

Figure 2 shows the time spent in each physical activity intensity distribution (<160 mg in Panel A and >160 mg in Panel B) by sex and age. There was a significant age and sex effect on intensity distributions (Wilks’ lambda = 0.06, *p* < 0.01). Girls were spending more time than boys in some lower intensities of activity (<30 mg), and boys were spending more time than girls in most intensities >70 mg, even when controlling for age (*p* < 0.05). There were also significant differences in time spent in each intensity of physical activity by age when controlling for sex (*p* < 0.05); as age increased, time spent in intensity of activity <40 mg was lower, and time spent in intensity >70 mg was higher. In all cases, the trend was for boys to spend more time in higher intensity physical activity and less time in lower intensity activity; and for time spent in higher intensity activities to be higher in the older age groups. When controlling for BMI z-score, weight or length, these relationships did not change (data not shown).

There was no significant interaction between developmental stage and sex (data not shown), however when dividing the data into developmental stage rather than age, boys spent more time in higher intensity activities than girls at each developmental stage, and both walkers and crawlers spent more time in activities >50 mg than infants who were not yet mobile.

**Fig. 3:**
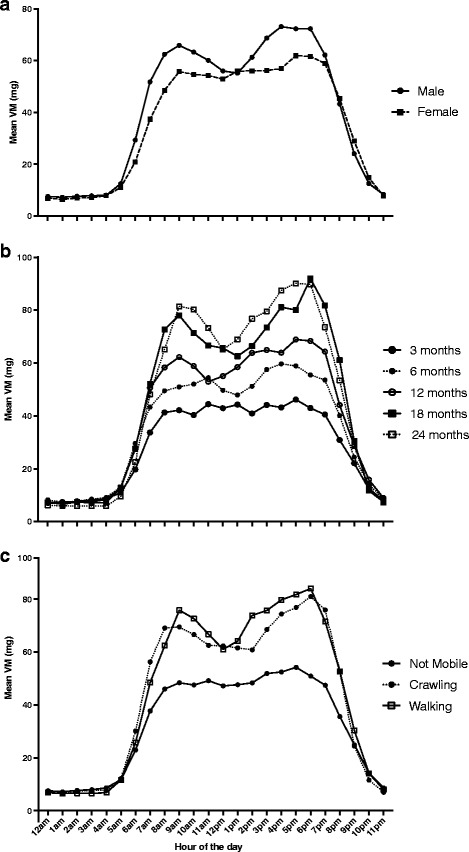
Mean diurnal distributions of activity by sex (**a**), age (**b**), and developmental stage (**c**)

Figure 3 shows the diurnal patterns of activity over an average 24-h period between boys and girls (Panel A), age groups (Panel B), and developmental stages (Panel C), respectively. Although patterns of activity were similar, boys had higher mean activity than girls, and this was significant at each hour between 6 am–11 am, and again from 3 pm–6 pm (*p* < 0.05), when controlling for age. Furthermore, when controlling for sex, as age increased, mean activity was higher and this was significant between 7 am-8 pm (*p* < 0.01). Between 2 am and 4 am, 3-month infants were significantly more active than 24-month infants (*p* < 0.05). By visual interpretation of the data, as well as through examining these statistical effects, it seems as if most infants were becoming active at 6 am and decreasing activity from about 9 pm. Younger infants seem to experience a short, 2-h active period between 2 and 4 am. Peaks in activity are seen around 9 am, and again at around 3 pm–6 pm with the highest activity accumulated between 5 pm–6 pm. There was a significant interaction between age and sex on physical activity intensity distribution throughout the day (Wilks’ Lamba = 0.20, *p* < 0.01). These relationships remained largely unchanged when controlling for BMI z-score (data not shown). Lastly, crawlers and walkers were more active during the day than infants who were not yet mobile, and in most cases walkers were more active than crawlers (except at 7 am, 12 pm, and 7 pm when crawlers were more active than walkers). There was a significant interaction between sex and developmental stage on distribution of physical activity intensity throughout the day (Wilks’ Lamba = 0.34, *p* < 0.01); and these relationships remained significant when controlling for age (Wilks’ Lambda = 0.13, *p* < 0.01), yet the effect of development was only significant at 7 am when crawlers were more active than walkers and not yet mobile infants.

Maternal reported time that infants spent in various activities (minutes per day) are shown in Table 2. Time spent outside was significantly higher in the older age groups and in boys (interaction effect: F = 3.84, *p* = 0.02). Simple main effects showed that boys spent more time outside than girls at all ages except 12-months, and that this effect was significant at 18-months (mean(SD) time outside per day was 135(88) minutes in boys and 45(19) minutes in girls (*p* = 0.01). Age effects were also seen for time spent restrained in a high chair or in a cot while awake, which was lower as age increased (*p* < 0.05); and time spent playing, either in a play area or alone, which was higher as age increased (*p* < 0.01). The majority (94%) of infants were exceeding TV time recommendations (no TV time recommended according to international guidelines [33, 34]). This value improved with age from 3% meeting guidelines at 3-months to 17% meeting guidelines at 18-months, and was not different by sex.

**Table 2:** Maternal reported infant activities by age

Infant activity (min/day)	3mo (*n* = 32)	6mo (*n* = 31)	12mo (*n* = 31)	18mo (*n* = 18)
Active time				
Total time free to play	60 (25–93)	90 (45–130)	150 (120–240)	158 (115–210)
*Games with an adult*	20 (5–30)	15 (2–45)	10 (5–30)	18 (4–30)
*Physical activity with mother*	8 (4–30)	10 (5–30)	15 (5–60)	18 (10–45)
*Tummy time*	20 (1–30)	10 (3–20)	NA	NA
*With older babies*	25 (7–75)	30 (10–120)	30 (15–120)	60 (40–120)
*Outside*	0 (0–0)	0 (0–30)	30 (0–60)	60 (50–120)
*With babies of same age*	0 (0–0)	0 (0–2)	0 (0–15)	28 (0–60)
*Play area*	0 (0–30)	30 (10–60)	60 (30–120)	60 (30–90)
*Floor play*	0 (0–25)	30 (5–60)	60 (30–90)	60 (30–120)
*Playing alone*	30 (15–60)	30 (10–45)	60 (30–60)	30 (20–30)
Restrained/sedentary time				
Total time restrained	133 (58–233)	150 (80–270)	100 (50–175)	75 (30–140)
*Pushchair*	0 (0–0)	0 (0–10)	0 (0–30)	0 (0–0)
*Baby chair*	0 (0–0)	10 (0–60)	NA	NA
*High chair*	0 (0–20)	20 (0–60)	10 (2–30)	10 (4–20)
*Car chair*	20 (3–60)	30 (5–30)	30 (10–60)	35 (15–60)
*Cot*	60 (30–120)	40 (20–90)	30 (15–60)	10 (5–30)
*Strapped to back*	30 (8–60)	30 (20–60)	30 (15–30)	NA
TV time	30 (20–90)	30 (10–90)	30 (5–45)	25 (10–60)

Values are median(IQR). Data not available for 24-months

*NA* Indicates that the activity was not asked about at the specific time-point

When controlling for age and sex in the combined sample, overall TV time trended towards a positive association with BMI z-score (β=0.01, *p* = 0.05). Estimated regression margins suggest a dose response relationship between TV time and predicted BMI z-score, with only 0 min TV time predicting a negative BMI z-score. Meeting TV time guidelines was also associated with developmental stage (*p* = 0.02), where a larger proportion of crawlers (18%) met the guidelines than walkers (2%) or not yet mobile infants (4%). There was a significant interaction effect for age and development on meeting TV time guidelines (*p* = 0.04), yet age and development were not independently associated.

Pairwise correlations were run separately by age for maternal reported variables and objectively measured mean infant physical activity. At 6-months, playing games with an adult (*r* = −0.40, *p* = 0.03), and time in a cot (*r* = −0.47, *p* < 0.01) were both inversely associated with objectively assessed infant physical activity. When stratifying by sex, at 12-months, lower maternal concerns about floor play were positively associated with physical activity for girls only (*r* = 0.54, *p* = 0.03). There were no significant correlates with objective physical activity at 3-, 18-, and 24-months; and mother’s age, maternal beliefs about physical activity (except floor play concerns at 12-months in girls); and maternal reported infant tummy time, time restrained, time free, or time in bed were not correlated with objective physical activity at any age.

## Discussion

This study is the first to objectively measure and describe physical activity levels and diurnal patterns in infants under age two in South Africa. Furthermore, few studies globally have thoroughly explored diurnal patterns and correlates of infant activities and behaviours in the first two years of life. We found that boys were significantly more active than girls throughout the day, regardless of age or developmental stage. These differences were evident as early as 3-months, and physical activity intensity increased significantly with age (regardless of developmental stage). Very few maternal or home environment variables were significantly associated with infant physical activity, yet it did seem that mothers provided boys with more opportunities to be active (eg. 18-month boys spending 135 min playing outdoors compared to 18-month girls spending only 45 min). Importantly, almost all of the infants were exceeding TV time recommendations.

To our knowledge, this is the first study to show sex differences in physical activity intensity and diurnal patterns from such an early age, although these differences are widely reported in older children and adults [24, 35]. Various studies conducted internationally have found no differences in objectively measured physical activity between boys and girls at approximately 24-months [36–40], however it is important to note that none of these studies explored physical activity intensity distributions; and only three explored diurnal patterns of physical activity – one in 19-month toddlers in Australia [39], one in 6- to 23-month infants/toddlers from Burkino Faso with moderate acute malnutrition [40], and one in Ethiopian toddlers with severe acute malnutrition in which sex differences were not reported [15]. The Australian study found that boys were more active than girls in the morning, but observed no overall sex differences [39]. The Yameogo et al., study found no differences between boys and girls [40], yet it is difficult to compare these results, since morbidity was found to be associated with activity levels in the children with malnutrition, and findings therefore cannot be extrapolated to a healthy population of infants. Most of these above mentioned studies that showed no sex differences had reported activity levels according to intensity cut-offs that have only been validated in older children and may not be appropriate in this age group. A recent systematic review of predictors of change in physical activity in 0–6 year olds found evidence that boys may be more active than girls, yet this finding was not consistent and was not shown as early in life as in the current study [14]. The sex differences observed in the current study are intriguing, as they beg the question whether boys are inherently more active than girls, starting almost from birth, or whether these differences are dependent on differential opportunities provided to boy infants compared to girls. These sex differences were found regardless of age or developmental stage, and remained significant after controlling for body size. Furthermore, physical activity was not associated with any maternal reported variables, with the exception of maternal reports of time spent outdoors. Mothers of boy infants reported their child spent more time outside than girl infants, yet this difference was only significant at 18-months of age. Since 18-months is the age at which the most notable differences in physical activity between boys and girls were shown, it is likely that by providing boy infants with more opportunities to be outside where children are known to be more active [41]- either due to cultural or social norms, or perceived ability- mothers are indirectly influencing sex differences in physical activity. Berglind et al., recently found that, in a group of four year old toddlers in Sweden, diurnal patterns of activity were different between boys and girls – with girls being less active and more sedentary than boys particularly when at home with parents, yet this difference was not observed during pre-school hours [42]. This finding could lend support to the theory that mothers provide boys with more opportunities to be active than girls. Perhaps this relationship is bidirectional or part of a positive feedback loop, particularly for older infants who have more autonomy around their behaviour decisions - where boy infants may appear to enjoy activities outside more than girls (eg: crying less when outdoors), thus providing positive feedback cues to the mother who in turn reacts by providing more time outside. By being provided with more opportunities to be active, boys may then be presenting with higher activity levels at later stages due to tracking and/or improved motor development.

We also showed differences in physical activity levels and patterns between different developmental stages, as well as age groups. These findings are not surprising, although it is important to note that age differences were found regardless of developmental stage and vice versa. Specifically, time spent in higher intensity physical activities was higher with age. Independent of this relationship, while both walkers and crawlers had higher intensity activity than not yet mobile infants; walkers had the highest intensity activity and this was mostly consistent throughout the day. There was an age effect introduced during the construction of the developmental stages, since questions about walking and crawling were not asked at 3-months (and these infants were assumed to be not yet mobile), while questions about walking were not asked at 6-months, and these infants were assumed to be not yet mobile if mothers stated they were not yet crawling. Furthermore, at 18 and 24-months, all infants were assumed to be walking. These assumptions, although valid, limit the conclusions that can be drawn; and the small number of infants reported to be crawling at 6-months and walking at 12-, and 18-months (less than one third in all cases) is concerning in comparison to international norms [32]. However, since the associations between developmental stage and physical activity were significant even after adjusting for age, they are worth considering in future studies. Similar to the current findings, walkers and crawlers were found to have higher activity (even after controlling for carrying) than not yet mobile infants in the Yameogo et al., study; yet the higher activity we observed in crawlers compared to walkers at certain times of day (7 am, 12 pm, and 7 pm) was not observed in the Yameogo et al., study [40]. The placement of the accelerometer on the wrist in the present study may have resulted in more movement being detected during crawling when the wrist is involved, than during walking. However, since walkers were still more active than crawlers at most times, there is potential that this finding could have also been affected by crawlers being more likely to be carried than walkers, thus limiting their opportunity for movement. Age at commencement of walking has been found to be associated with higher physical activity levels in toddlers in Australia, regardless of how long the toddler had been walking when examined, suggesting an innate effect of early motor development on physical activity levels [19]. Furthermore, earlier infant motor development has been associated with greater sports participation at age 14, suggesting a tracking effect [21]. The current findings are thus important, since it appears that, besides physical activity intensity increasing with age, early attainment of developmental milestones is important for both contemporary and future physical activity. Furthermore, providing opportunities to be active in early infancy (such as tummy time), has been associated with earlier attainment of developmental milestones [43] – suggesting a bidirectional effect.

Recent evidence has suggested that physical activity levels in young children differ over the course of the day [39, 42, 44]. The present study has confirmed that infant levels of physical activity follow a similar pattern of accumulation to those observed in older children over the course of the day. Diurnal patterns were able to indicate when infants were accumulating the most activity during the day. The peak in activity seemed to occur between 4 and 6 pm – tending more towards 4 pm in the younger infants and more towards 6 pm in older infants (possibly due to more engagement with family members or opportunities for active play). Smaller peaks occurred around 9 am, with slumps occurring between 12 and 1 pm (likely due to feeding times and/or napping in the younger infants). These patterns are very similar to those observed in 19-month toddlers in Australia, where peaks were seen between 8 and 10 am, and between 4 and 5 pm, with slumps observed at midday [39]. These diurnal patterns are helpful for the understanding of natural, habitual activity behaviours in infants, which would allow for optimisation of intervention design. Furthermore, understanding normal infant/toddler behaviours allows for comparisons with other infant/toddler populations and may provide a means to determine alterations in normal behaviour signifying illness or other issues. For example, a study which similarly looked at activity patterns in 6–23 month infants/toddlers with severe acute malnutrition, found similar waking times and peaks around 9 am, yet found peak activity to occur quite a bit later than in the current study [40]. This could be a cultural or sample specific effect and relationships with health are not clear, yet is worth noting since comparative data in this field is so limited. The authors also found that younger infants were more active than older toddlers during awake hours – which is the converse of the findings from the current study, and indicates shifts in normal behaviours, likely in order to conserve energy for brain and body growth during a period of severe malnutrition [45]. Indeed, Ketcheson et al. recently concluded that examination of daily patterns in physical activity according to age and development in infants are warranted in order to understand differences that may occur in special populations (such as infants with Down’s Syndrome) [46].

Since there are no defined physical activity cut-offs for different intensities of activity in infants (although cut-offs for two year olds have been developed [47] these could not be extrapolated to such young infants who are not yet mobile), we examined the entire distribution of physical activity across a broad range of intensities up to 400 mg (where very little time was spent in this sample). This allowed identification of the movement intensities where age and sex differences appear to be greatest, around the 60-70 mg intensity threshold. Using transparent data processing made possible with Axivity AX3 monitors, and describing distribution intensities rather than categorising data according to cut-offs, provides a preliminary movement growth curve against which individual children can be compared. However, further work involving either direct observation or a gold standard measure of energy expenditure would be required to understand what may define high or low intensity activities in infants under age two.

There were very few associations between objectively measured physical activity and maternal reported factors and maternal beliefs. This is the first study to report on maternal beliefs about physical activity and maternal reported activities in infants under two in South Africa. Unlike a study done in 19-month toddlers from Australia [19] that used the same questionnaires as this study, neither time spent with other children, or time spent being active with mother were associated with objectively measured physical activity. In fact, in the present study time spent playing games with an adult was associated with lower physical activity at 6-months of age. This is possibly due to the types of games being played in this age group and setting, which may include sedentary activities such as reading (which are still cognitively beneficial but do not produce high intensity movements). Further qualitative work is thus warranted to contextualise types of activities common in this setting.

Overall time spent restrained was high (average of 161 min/day for the whole sample), but showed an inverse relationship with age. These findings are logical – as infants become independently mobile, parents are likely to keep them restrained less often. Conversely, overall time spent free to play was low (average 138 min/day for the whole sample), but showed a direct relationship with age (a similar trend as observed with objectively measured physical activity). These findings are similar to those from an Australian sample of infants, where more unrestricted time was reported with age (60 min/day in both Australian infants at 4-month and in South African infants at 3-months, and 180 min/day in Australian 9-month infants vs 150 min/day in South African 12-month infants) [48]. However unrestricted time was much higher in the Australian toddlers at 20-months (420 min/day) compared to the South African toddlers at 18-months (158 min/day). Overall restricted time was very similar in the Australian study to those reported in the present study (133 min/day at 4-months in Australia vs 119 min/day at 3-months in South Africa; 150 min/day at 9-months in Australia vs 154 min/day at 12-months in South Africa; and 158 min/day at 20-months in Australia vs 150 min/day at 18-months in South Africa). Interestingly, Australian mothers reported that their infants/toddlers spent much more time being active with a parent than did the South Africa mothers (43 min/day at 4-months vs 8 min/day at 3-months; 60 min/day at 9-months vs 15 min/day at 12-months; and 60 min/day at 20-months vs 18 min/day at 18-months in Australia vs South Africa respectively) [48]. The patterns shown in the current study suggest that the infants who were offered the most opportunity to be active, were also those who spent the most time in higher intensity activities; but also that South African mothers may not be engaging in enough active play with their infants or providing enough opportunity to be active in comparison to international data.

Only one maternal belief was associated with physical activity: lower concerns about floor play were associated with higher physical activity at 12-months for girls only, suggesting that mothers who were less concerned about their infant being left free to move around on the floor may have provided more opportunity to do so, resulting in higher levels of activity. The fact that this association was only observed for girl infants again points to differential sex specific maternal behaviours and beliefs resulting in providing increased opportunities for physical activity for boy infants, in conjunction with the existence of enhanced safety concerns for girl infants; which could either be due to inherent beliefs or feedback from infant behaviours. Most maternal beliefs about activity were not associated with objectively measured physical activity. It is possible that the questions used in this measurement tool were not well understood in this sample, even though they were piloted and adapted for the Soweto context before use, and internal consistency was acceptable (mean Cronbach alpha = 0.60). It is also possible that maternal beliefs about physical activity are not necessarily translating into practice due to constraints of the environment, time availability and family needs, or other barriers that may be preventing mothers from providing sufficient opportunity for play in the Soweto area. Indeed, previous studies in Soweto have shown that young adults report high levels of alcohol and drug abuse, crime, illegal dumping of waste, sewerage issues, road rage, and gang presence [49]– all of which could influence perceived safety for activities such as outdoor play. Furthermore, in a survey of young mothers in Soweto, most mothers lived in households with more than five occupants, and cared for children other than their own biological children, and the majority did not live with the father of their child [22]. These potential barriers definitely require further investigation, in order to better understand the context of these beliefs, and consequent behaviours in such a unique environment.

Most (90%) infants were meeting the recommended 180 min of active time as reported by mothers. However, 94% of infants were not meeting international TV time recommendations of zero minutes of TV time in children under age two [33, 34], and mothers reported on average 50 mins in front of the TV per day. TV time was lower in older infants, which, when taken in conjunction with the increased time spent outdoors and higher levels of physical activity in older infants, may be an indication that older infants were substituting TV time with outdoor time and active play. It must be noted that using time in front of a TV as a proxy for TV time has limitations, including the inability to dissociate activities which may be occurring simultaneously (such as sitting in front of the TV whilst playing on the floor – which may be better categorised as active time). Nonetheless, exceeding TV time recommendations using this proxy variable was associated with lower motor milestone attainment, and crawlers were the most compliant with guidelines (18% meeting guidelines) compared to walkers (2% meeting guidelines) and not yet mobile infants (4% meeting guidelines). When treated as a continuous variable, minutes spent watching/in front of the TV was directly associated with BMI z-score in the whole sample, with each 10 min spent in front of the TV being associated with approximately +0.05 in BMI z-score. This is similar to recent findings from the UK, where 75% of infants exceeded recommendations [50], and TV time was associated with increased BMI.

This study has several limitations. Firstly, the cross sectional nature of the data limits our ability to determine causality. Furthermore, the assumptions that were made in order to categorise the developmental stages require that the data be interpreted with caution. Some data were not collected at every age (i.e.: maternal beliefs data not collected at 3-, 18-, and 24-months; development and maternal reported physical activity not collected at 24-months), resulting in missing data for these questions. Furthermore, the sample size was small when stratifying by age; and findings cannot necessarily be extrapolated to infants outside of South Africa. Lastly, the inability to differentiate between caregiver carrying movements and infant initiated movement limits the interpretation of some of the findings around carrying and objectively measured physical activity, and future work should examine methodological means to deal with this confounding effect. However, the strengths of this study lie in the use of objective measurement of raw acceleration data, which allows full control and transparency of data processing. By examining the distributions of activity across all intensities, we were able to interpret associations that may have been lost if the data were categorised into broader intensity categories.

## Conclusions

In conclusion, this study is the first to show sex differences in the patterns and intensity of physical activity in the first two years of life. Total physical activity was higher in boys, older age groups, and in those with more advanced developmental stages. Children who were provided more opportunities to be active, either through floor play or playing outside, achieved higher levels of activity. Understanding these patterns of activity and the factors associated with them is the first step in being able to determine when and how to intervene effectively. While maternal reported time spent free to be active was sufficient as reported by mothers, and meets minimum recommendations; most infants were spending excessive time in front of a TV, and this TV time was inversely associated with BMI z-score. Thus, providing opportunities to be active through play, and limiting screen time to zero should be encouraged.

## Abbreviations

BMI: Body mass index
CDC: Centre for Disease Control
TV: Television
WHO: World Health Organization
